# Endothelial MAPKs Direct ICAM-1 Signaling to Divergent Inflammatory Functions

**DOI:** 10.4049/jimmunol.1600823

**Published:** 2017-04-03

**Authors:** Silvia Dragoni, Natalie Hudson, Bridget-Ann Kenny, Thomas Burgoyne, Jenny A. McKenzie, Yadvinder Gill, Robert Blaber, Clare E. Futter, Peter Adamson, John Greenwood, Patric Turowski

**Affiliations:** Department of Cell Biology, Institute of Ophthalmology, University College London, London EC1V 9EL, United Kingdom

## Abstract

Lymphocyte transendothelial migration (TEM) is critically dependent on intraendothelial signaling triggered by adhesion to ICAM-1. Here we show that endothelial MAPKs ERK, p38, and JNK mediate diapedesis-related and diapedesis-unrelated functions of ICAM-1 in cerebral and dermal microvascular endothelial cells (MVECs). All three MAPKs were activated by ICAM-1 engagement, either through lymphocyte adhesion or Ab-mediated clustering. MAPKs were involved in ICAM-1–dependent expression of TNF-α in cerebral and dermal MVECs, and CXCL8, CCL3, CCL4, VCAM-1, and cyclooxygenase 2 (COX-2) in cerebral MVECs. Endothelial JNK and to a much lesser degree p38 were the principal MAPKs involved in facilitating diapedesis of CD4^+^ lymphocytes across both types of MVECs, whereas ERK was additionally required for TEM across dermal MVECs. JNK activity was critical for ICAM-1–induced F-actin rearrangements. Furthermore, activation of endothelial ICAM-1/JNK led to phosphorylation of paxillin, its association with VE-cadherin, and internalization of the latter. Importantly ICAM-1–induced phosphorylation of paxillin was required for lymphocyte TEM and converged functionally with VE-cadherin phosphorylation. Taken together we conclude that during lymphocyte TEM, ICAM-1 signaling diverges into pathways regulating lymphocyte diapedesis, and other pathways modulating gene expression thereby contributing to the long-term inflammatory response of the endothelium.

## Introduction

Transendothelial migration (TEM) of leukocytes is well coordinated and occurs during inflammation and homeostatic immune surveillance of tissues. Initially, leukocytes adhere loosely to the vascular wall and roll along its luminal surface before being arrested by more firm adhesive interactions. They then undergo diapedesis—the penetrative phase of passage through the endothelial cell (EC) barrier (1). ICAM-1 and VCAM-1 on the surface of ECs are key in mediating firm adhesion of leukocytes. ICAM-1 acts as a gatekeeper of lymphocyte TEM (2) by binding to activated β_2_ integrins, namely LFA-1 (α_L_β_2_; CD18/CD11a). Following engagement, ICAM-1 redistributes to perijunctional areas (3), which are also the sites of active diapedesis (4).

ICAM-1 also initiates outside-in signaling that facilitates diapedesis, increases vascular permeability, and regulates the endothelial inflammatory response (2, 3, 5, 6). Endothelial signaling downstream of ICAM-1 involves Rho GTPases, intracellular calcium (Ca^2+^), endothelial NO synthase, actin cytoskeletal rearrangements, protein kinase C (PKC) and Src family kinases. Many of these components of ICAM-1 signaling are indispensable for lymphocyte TEM, both in vitro and in vivo (7–11). For the current study, an important downstream effect of endothelial ICAM-1 activation is phosphorylation of the adherens junction (AJ) component vascular endothelial cadherin (VE-cad; cadherin 5), which is mediated via Ca^2+^ and NO (3, 10, 12). In microvascular ECs (MVECs) from the brain, ICAM-1 cross-linking also leads to phosphorylation of the cytoskeletal scaffold protein paxillin via a Rho-dependent pathway, which suggests a potential role for this protein during lymphocyte TEM (7). Indeed, paxillin has recently been shown to be required for neutrophil TEM across HUVECs (13). A plethora of protein kinases, including focal adhesion kinase (FAK), Src, and MAPKs, can phosphorylate paxillin on multiple sites (14). Phosphorylation of Y31 and Y118 by FAK or Src are key for paxillin function and its interactions with other proteins.

ICAM-1 activation also increases inflammatory gene expression, e.g., that of *IL-1B* (15), *CXCL8* (*IL-8*), *CCL5* (*RANTES*) (16), and *VCAM-1* (17), although it is unclear how, or if, this relates to leukocyte TEM.

The MAPKs ERK, p38, and JNK regulate numerous cellular processes, including gene expression, cell survival, and cell motility (18). As such, MAPKs mediate gene expression in ECs at various levels (19). In addition, p38 controls posttranscriptional stabilization of mRNAs that have AU-rich regions in their 3′ untranslated region, including transcripts for TNF-α and cyclo-oxygenase 2 (COX-2) (20). All three endothelial MAPKs are activated in response to ICAM-1 engagement, although the implication of this in relation to TEM of lymphocytes is unclear. To this end, it has been shown that ERK mediates ICAM-1–induced expression of VCAM-1 in HUVECs (17). In brain MVEC, activation of JNK in response to ICAM-1 engagement occurs via a Rho-dependent pathway (7), which implies a potential role in supporting TEM of lymphocytes. Finally, in TNF-α–stimulated pulmonary MVECs, ICAM-1 engagement induces activation of p38 leading to actin cytoskeletal rearrangements and phosphorylation of heat shock protein 27 (21).

We hypothesized that MAPKs are key regulators of diapedesis-related and nondiapedesis-related processes during ICAM-1–mediated TEM and investigated the roles of ERK, p38, and JNK in response to ICAM-1 activation in cerebral and dermal MVECs. We report that ERK, p38, and JNK were all involved in modulating inflammatory gene expression. In contrast, TEM of lymphocytes was mainly controlled by JNK, which in cerebral MVECs promoted phosphorylation of paxillin and its subsequent interaction with VE-cad.

## Materials and Methods

### Materials

Gö6983, PP2, Mowiol 4-88, SB202190, and SP600125 were purchased from Merck (Nottingham, U.K.); cell-permeable C3 transferase was from Universal Biologicals (Cambridge, U.K.); PF573228 and FAK inhibitor 14 were from Tocris (Bristol, U.K.); U0126 was from Promega (Southampton, U.K.); L-JNKi was from Alexis Biochemicals (Exeter, U.K.); actinomycin D, myelin basic protein (MBP), and anti-tubulin ascites (clone DM1A) were from Sigma (Poole, U.K.). Mouse anti-rat CD54 (clone 1A29), mouse anti-human CD54 (clone 15.2), anti-rat CD18 (clone WT3), anti-rat CD11a (clone WT1), and anti-rat CD49d were from AbD Serotec (Kidlington, U.K.). GST c-Jun was purchased from Cell Signaling Technology (Hertfordshire, U.K.) and GST-ATF2 from Upstate Biotechnology (Lake Placid, NY). Abs against phosphorylated and total ERK, JNK, p38, and paxillin (Y118) were all from Cell Signaling Technology (Hertfordshire, U.K.). Anti-phosphotyrosine (clone 4G10) was from Millipore (Watford, U.K.) and anti-paxillin from BD Transduction Laboratories (Oxford, U.K.). Affinity purified rabbit anti–VE-cad has been described previously (22). Anti–VCAM-1 Abs were from Santa Cruz Biotechnology (sc-1504). Additional reagents for indirect cell immunochemistry included fluorescein-conjugated goat anti-rabbit IgG (MP Biochemicals, Cappel), Cy3-conjugated goat anti-mouse IgG (Jackson ImmunoResearch), and Zenon Alexa Fluor 546 Mouse IgG_1_ (Invitrogen, Paisley, U.K.). HRP-coupled Abs for immunoblots were from GE Healthcare (Buckinghamshire, U.K.).

### EC culture

ECs were cultured on tissue culture plasticware (Nunc, Roskilde, Denmark) coated with polymerized collagen I (BD Biosciences, Oxford, U.K.). The immortalized rat brain MVEC line GPNT was maintained as previously described (10). Primary cultures of rat brain MVECs (23) and the human brain MVEC line hCMEC/D3 (24) were cultured in EGM2-MV medium (Lonza, Slough, U.K.). Human dermal MVEC (PromoCell) were cultured on tissue culture plasticware coated with gelatin (Sigma) and maintained in EC growth media MV2 (PromoCell). Quality of EC cultures was routinely assessed by visual inspection and transendothelial electrical resistance (TEER) measurements (23). Importantly, none of the treatments (pharmacological antagonists or transfections) had any effect on EC monolayer integrity.

### Lymphocyte coculture, adhesion, and TEM assays

Three different types of lymphocyte were used. Peripheral lymph node cells (PLNCs) were isolated from cervical or juxtaintestinal lymph nodes of Wistar or Lewis rats (Olac, Harlan) (3). PLNC cultures consist predominantly of nonantigen-activated lymphocytes, which do not display significant TEM rates within 4 h of coculture with brain MVECs (25). The migratory MBP-specific rat T cell line (PAS; a kind gift from Dr. E. Beraud, Marseille, France) consists of clonal MHC class II–restricted CD4^+^ cells (26) that also express IFN-γ (Supplemental Fig. 3A) and can thus be classified as Th1. Human CD4^+^ cells were isolated from peripheral blood using Ficoll Paque, and positive selection on CD4^+^ MACS beads (Miltenyi, Bisley, U.K.) (10).

For rat brain MVEC monolayer stimulation and adhesion assays, PLNCs, activated with 5 μg/ml Con A in EC-conditioned, serum-free growth medium, and optionally fluorescently labeled with 1 μM calcein-AM (Invitrogen), were used (3). For immunoblot analyses, PLNCs were added at a 5:1 ratio to EC monolayers. At various times adherent PLNCs were extensively washed off with ice-cold PBS before endothelial lysis to restrict studies to ECs (visual inspection showed approximately one PLNC remaining per 250 ECs). For some adhesion assays lymphocyte cell adhesion molecules were neutralized using Abs. For this 1 × 10^7^ PLNCs were incubated on ice for 1 h with 20 μg/ml of anti–adhesion molecule Ab. Neutralization of LFA-1 was performed using function-blocking Abs against CD18 and CD11a. This approach affects all β_2_ integrins (CD18). However, CD18/CD11a is the only β_2_ integrin expressed on lymphocytes; the other three β_2_ integrins are only found in myeloid cells (27). Function-blocking Abs against CD49d were used to neutralize VLA-4.

Migratory lymphocytes (rat PAS or human CD4^+^) were cultured at 1 × 10^6^/ml in RPMI 1640 supplemented with 10% FCS, 100 U/ml penicillin, 100 μg/ml streptomycin, 1 mM sodium pyruvate, 1 mM nonessential amino acids, 2 mM l-glutamine, and 50 μM β-mercaptoethanol in the presence of IL-2 (50 U/ml). TEM assays were performed by time-lapsed video microscopy exactly as previously described (3, 10).

### ICAM-1 ligation, cross-linking, and small molecule inhibitor pretreatment

ICAM-1 ligation or cross-linking was performed as previously described (10). To study endothelial MAPK involvement, postconfluent, serum-starved GPNTs were pretreated for 1 h with 50 μM U0126 (an inhibitor of the ERK-activating kinase MEK), SP600125 (an inhibitor of JNK), and SB202190 (an inhibitor of p38α and β) and then washed extensively. Due to the presence of efflux pumps on brain MVECs, the extracellular concentration of inhibitors had to be relatively high to ensure MAPK activity was significantly suppressed (Supplemental Fig. 2B–D).

### Immunoblotting and protein kinase assays

Cell lysates were prepared as previously described (3). Proteins were electrophoretically separated on SDS-polyacrylamide gels and immunoblotted (3, 10).

To assess intracellular MAPK activity, cells were lysed in ice-cold lysis buffer containing 50 mM Tris-HCl pH 7.4, 150 mM NaCl, 50 mM NaF, 10 mM β-glycerophosphate, 0.1 mM EDTA, 100 nM calyculin A, 1 mM PMSF, 1 mM sodium orthovanadate, and 2 μg/ml each of pepstatin, aprotinin, and leupeptin. Equal amounts of clarified lysates were then incubated with 1 μg anti-ERK, anti-JNK, or anti-p38 Ab and protein G–Sepharose beads. Immunocomplexes were washed three times with lysis buffer and further processed for kinase assays in 30 μl reaction volumes containing 25 mM HEPES pH 7.5, 10 mM magnesium acetate, 5 μM of cAMP-dependent protein kinase peptide inhibitor (PKI), 100 μM [γ-[^32^P]]ATP (1000 Ci/mol), and 1–5 μM of MBP, GST-c-Jun, or GST-ATF2 for ERK, JNK, or p38, respectively. The mixture was incubated at 30°C for 5–10 min, stopped with 30 μl of 2× Laemmli sample buffer, boiled, and then analyzed by SDS-PAGE and autoradiography. The amount of immunoprecipitated kinase was determined by immunoblotting.

### RT-PCR and RNA stability analyses

Total RNA from GPNTs or PAS was prepared using the RNeasy kit (Qiagen, Crawley, U.K.). Total RNA (0.25 μg for GPNT; 1 μg for PAS) was reverse transcribed using Superscript III (Invitrogen). PCR reactions were performed using 1 μg of cDNA and sequence-specific primers as detailed in Supplemental Table I. Preliminary dose-response reactions ensured that detection of each transcript occurred in the linear range of amplification (data not shown). PCR products were separated by agarose gel electrophoresis, stained with ethidium bromide, and acquired with GeneSys software (Syngene). The m.w. of the PCR product was compared with the 100 bp DNA ladder (New England BioLabs).

For RNA stability analyzes, total RNA was isolated from GPNT cells at various time points following transcriptional blockage through actinomycin D treatment (10 μg/ml). Total RNA (1 μg) was DNase-treated and reverse transcribed using a Quantitect Reverse Transcription Kit (Qiagen). For TNF-α, cDNA was preamplified using Taqman PreAmp Mastermix (Applied Biosystems, Paisley, U.K.) and RT-PCR was performed using Taqman Gene Expression Assays (Assay ID Rn99999017_m1) according to the manufacturer’s instructions (Applied Biosystems). COX-2 and ribosomal protein were analyzed using Power SYBR Green PCR Mastermix (Applied Biosystems). All reactions were run on an ABI7900HT RT-PCR machine and results analyzed using DART-PCR software.

### ELISA and multianalyte flow assay

MVEC supernatants were collected after 4, 8, 12, 24, or 48 h of ICAM-1 cross-linking. ELISA was performed using the LEGEND MAX Human TNF-α ELISA Kit with Precoated Plates (BioLegend) according to the manufacturer’s instructions. Multianalyte flow assay of chemokines relevant to neuroinflammation (28) was performed using the LEGENDplex Multi-Analyte Flow Assay Kit (BioLegend). Optionally anti–TNF-α Abs (1 μg/ml; R&D Systems, Abingdon, U.K.) were added throughout the ICAM-1 stimulation period.

### Plasmids, nucleofection, and luciferase assays

Plasmids encoding chicken (wild-type and dominant-negative Y31F/Y118F; kindly provided by Dr. R. Horwitz, University of Virginia) (29) and human paxillin (wild-type and S178A; kindly provided by Dr. K. Jacobson, University of North Carolina), FLAG-tagged JNK1, JNK2, and MKK7 (wild-type and dominant-negative; kindly provided by Dr. R. Davis, University of Massachusetts Medical School), and the pGL3-VCAM-1-promoter-luciferase plasmid construct (pVCAM-1-luc; kindly provided by Prof T. Minami, University of Tokyo, Japan) were nucleofected into subconfluent GPNTs at 10 μg of plasmid per 3 × 10^6^ ECs according to the manufacturer’s instructions (Amaxa, Cologne, Germany). Transfection rates, determined by indirect or direct fluorescent microscopy, were 60–80% except for pVCAM-1-luc, which transfected at a slightly lower efficiency of 30–50% (data not shown). Transfected cells were replated in full-growth medium and experimental analysis was carried out 48 h later (3). Luciferase activity was determined using a reporter assay system (Promega). All cells used in a single experiment were derived from a single transfection and the same number of cells was plated for each analysis point, which allowed direct comparison of luciferase activity between samples.

### Immunocytochemistry

Cells were fixed and processed for indirect immunocytochemistry of F-actin using Alexa Fluor 488 phalloidin (1:50; Invitrogen) or Abs against pan and phosphorylated paxillin (Cell Signaling Technology) as previously described (23). All immunostained preparations were further stained with 1 μg/ml bisbenzimide (33258; Hoechst), mounted using Mowiol 4-88 and analyzed on a LSM 700 confocal laser-scanning microscope (Carl Zeiss, Hertfordshire, U.K.). Images were acquired as 8-bit tif files and further processed using Adobe Photoshop CS5 or National Institutes of Health ImageJ 1.50c4.

### VE-cad endocytosis

VE-cad internalization was monitored by two independent methods essentially as described (30). For biochemical analysis live GPNT cells were stimulated or not with anti–ICAM-1 Abs. After varying times, they were washed three times with ice-cold PBS to stop any further endocytosis and then incubated on ice with freshly prepared trypsin (1 mg/ml) for 30 min. Reactions were stopped by the addition of soy bean trypsin inhibitor (50 mg/ml). Cells were harvested by centrifugation, lysed, and analyzed by immunoblot. Full-length, trypsin-resistant VE-cad was considered as internalized.

Alternatively, confluent hCMEC/D3 were labeled with FITC-conjugated anti–VE-cad Ab (Serotec rabbit anti-human CD144:FITC) on ice (to avoid its internalization) for 1 h. Cells were washed and returned to 37°C in the presence or absence of anti–ICAM-1 Abs. Cells were fixed with 3.7% formaldehyde, optionally after an acid wash in PBS, 25 mM glycine, 3% BSA, pH 2.7 to remove extracellularly bound Ab (and to reveal exclusively Ab bound to internalized VE-cad). Fixed cells were then processed for image acquisition by confocal microscopy (see above).

### Immunogold electron microscopy

hCMEC/D3 were fixed in 4% PFA and 0.1% glutaraldehyde and processed as previously described (23). Sections of 80 nm thickness were then stained using Abs against the extracellular domain (TEA 1.31; Serotech) or the C terminus (sc-6458; Santa Cruz) of VE-cad. Samples were viewed on a Jeol 1010 TEM, and images were gathered using a Gatan OriusSC100B charge-coupled device camera. Further image manipulation was performed in Gatan Digital Micrograph and Adobe Photoshop. VE-cad distribution was determined by visual inspection of electron micrographs. The distance of gold particles from interendothelial junction areas was measured using ImageJ.

### Data and statistical analysis

Densitometric quantification of at least three independent immunoblots were calculated and determined by changes in phosphoprotein content normalized to tubulin/total paxillin loading controls, with values expressed as fold increase. All numerical data were presented as mean ± SEM. TEM and adhesion data were expressed as percentage of control (TEM: mean ± SEM of six replicates from at least six independent experiments; adhesion: mean ± SEM of 12 replicates from three independent experiments). Statistics were performed using one-way ANOVA, using the SPSS Statistics 17 software, with significance levels set at 0.05, followed by Student *t* test (for pairwise comparison), Dunnett or Bonferroni post hoc analysis: ns, *p* ≥ 0.05; *, *p* < 0.05; **, 0.001 < *p* < 0.01, ***, *p* ≤ 0.001. Time-dependent RNA decay data were analyzed by linear regression and the significance of slopes determined by ANOVA: two-factor with replication.

## Results

### ICAM-1 engagement on brain MVECs leads to MAPK activation

**FIGURE 1.**
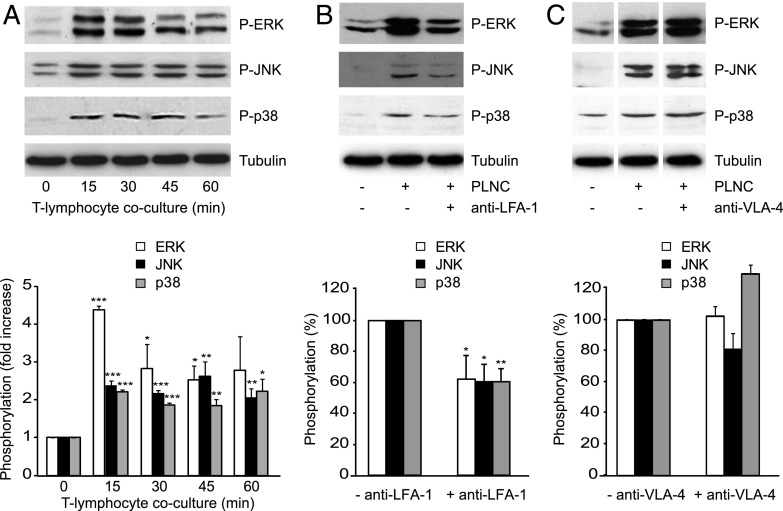
Endothelial MAPK activation in response to lymphocyte adhesion. (**A**) All three MAPKs were activated in GPNT ECs cocultured with Con A–activated, nonmigratory rat PLNCs. Shown are representative immunoblots of MAPKs phosphorylation alongside tubulin loading controls and normalized densitometric quantification of three independent experiments. (**B** and **C**) MAPK activation in GPNT in 30 min cocultures was reduced when PLNCs were preincubated with function-blocking anti–LFA-1 Abs but not an anti–VLA-4 blocking Ab. Shown are representative blots and densitometric quantification of three independent experiments. Control phosphorylation levels (in response to PLNC adhesion without adhesion molecule neutralization) were set to 100%. Data were compared with the corresponding time 0 controls and significant differences are indicated. In (C), white separation lines indicate where lanes from the same blots were joined. *p < 0.05, **p < 0.01, ***p < 0.001.

Adhesion-dependent endothelial MAPK activation was studied in cocultures of brain MVECs (GPNT) and Con A–activated PLNC that adhere but do not undergo diapedesis (25). At least a 2-fold increase in the amount of phosphorylated endothelial ERK, JNK, and p38 was observed within 15 min of coculture, and this response was sustained for at least 1 h (Fig. 1A). Ab-mediated neutralization of LFA-1, the principal ICAM-1 counter receptor on lymphocytes, reduced endothelial MAPK phosphorylation by at least 40% (Fig. 1B), whereas neutralization of VLA-4 had no effect on the increased phosphorylation of endothelial MAPKs (Fig. 1C).

**FIGURE 2.**
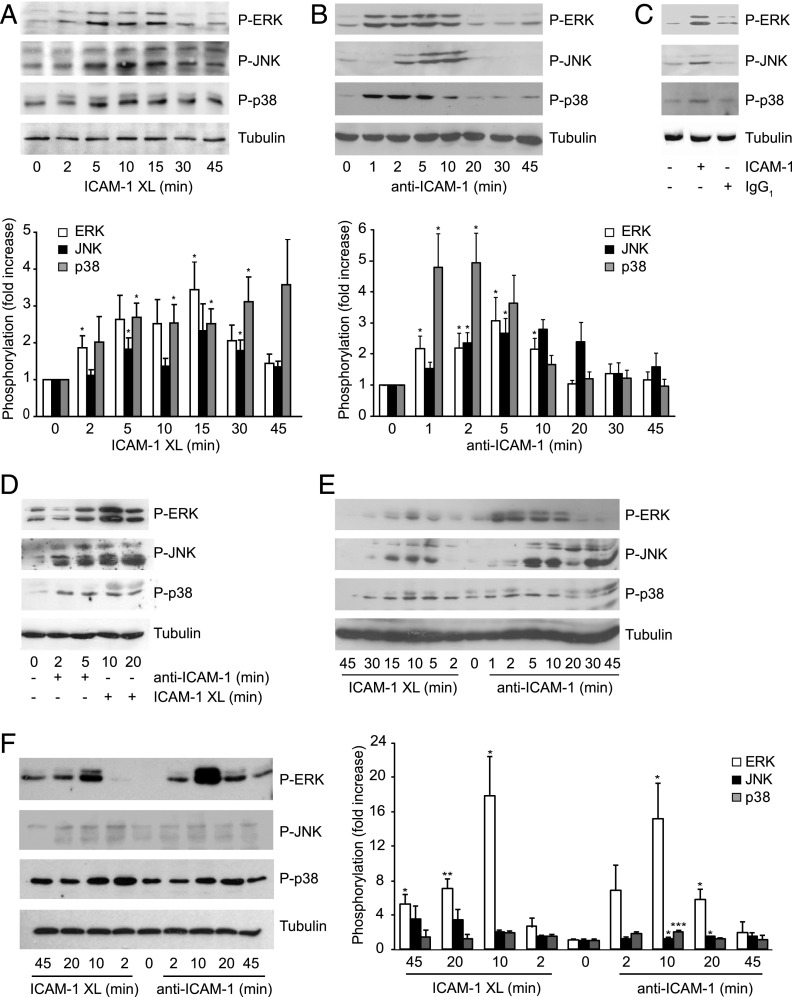
Endothelial MAPK activation in response to ICAM-1 ligation and cross-linking. (**A**) GPNT were subjected to ICAM-1 cross-linking (XL), with secondary clustering, for the indicated length of time and MAPK phosphorylation analyzed. Representative results and quantification of kinase activation (normalized mean ± SEM) from three independent experiments are shown. (**B**) Representative analysis of MAPK phosphorylation following ICAM-1 ligation (without secondary clustering) for the indicated times and densitometric quantification of kinase activation of three independent experiments. MAPK levels were not significantly affected by ICAM-1 ligation or cross-linking (Supplemental Fig. 1A, 1B). (**C**) Postconfluent, serum-starved GPNT cells were either left untreated or incubated with 5 μg/ml anti–ICAM-1 (1A29) or isotype-matched control IgG for 10 min. (**D**–**F**) Primary rat brain MVEC (D) or hCMEC/D3 (E) or human dermal MVEC (F) were stimulated by ICAM-1 cross-linking (XL) or ICAM-1 ligation for the indicated times and MAPK phosphorylation analyzed as described in (B). Data were compared with the corresponding time 0 controls and significant differences are indicated. *p < 0.05, **p < 0.01, ***p < 0.001.

Stimulation of ICAM-1 on its own by Ab-mediated cross-linking, which mimics the ICAM-1–relevant response of lymphocyte adhesion (10), also increased the level of phosphorylated MAPKs (Fig. 2A). This was not due to increased levels of each MAPK itself (Supplemental Fig. 1A). The phosphorylation of p38 was induced more rapidly and sustained for much longer than that observed for ERK and JNK. Incubation with anti–ICAM-1 Ab alone (in the absence of a secondary, cross-linking Ab) led to similar albeit more short-lived activation of MAPKs (Fig. 2B). Incubation with an irrelevant, isotype-matched Ab did not induce significant MAPK phosphorylation (Fig. 2C). Increased phosphorylation of endothelial MAPKs was also observed following stimulation of ICAM-1 on primary rat brain MVECs (Fig. 2D) or the human brain EC line hCMEC/D3 (Fig. 2E). MAPK kinases, in particular ERK, were also significantly activated in primary human dermal MVECs (Fig. 2F, Supplemental Fig. 1B), indicating that ICAM-1–induced MAPK activation was not restricted to the brain endothelium. For all further experiments ICAM-1 cross-linking was chosen to study long-term effects of MAPK signaling whereas simple ICAM-1 ligation was used to reproduce immediate early effects of ICAM-1 signaling.

### Endothelial MAPKs regulate ICAM-1–induced gene expression

**FIGURE 3.**
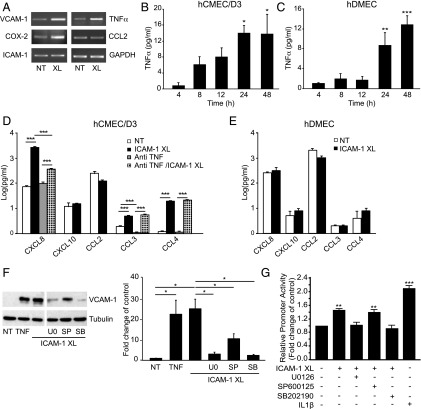
Role of MAPK in ICAM-1–mediated gene expression. (**A**) Total RNA was isolated from untreated (NT) or 4 h ICAM-1 cross-linked (XL) GPNTs and analyzed by semiquantitative RT-PCR for message levels of VCAM-1, TNF-α, COX-2, CCL2, ICAM-1, and GAPDH. (**B** and **C**) Confluent hCMEC/D3 (B) or human dermal MVEC (hDMEC) (C) were either left untreated or subjected to ICAM-1 cross-linking. At the indicated times TNF-α concentration in the culture supernatant was determined by ELISA. Shown are mean levels ± SEM of TNF-α above those in control cells from three independent experiments. (**D** and **E**) hCMEC/D3 (D) or human dermal MVEC (hDMEC) (E) were left untreated (NT) or subjected to ICAM-1 cross-linking (XL) for 24 h. The concentration of CXCL8, CXCL10, CCL2, CCL3, and CCL4 in the supernatant was measured by multianalyte flow assay. Where indicated anti–TNF-α (1 μg/ml) was included during the stimulation period to determine if altered chemokine secretion was a consequence of TNF-α induction. Shown are mean concentrations ± SEM of chemokines in the culture supernatant as determined from three independent experiments. (**F**) Confluent GPNT ECs were either left untreated (NT) or treated with 200 U/ml TNF-α for 12 h or subjected to ICAM-1 cross-linking (XL) for 12 h prior to immunoblot analysis of VCAM-1 and tubulin. Shown is a representative blot and densitometric quantification of three independent experiments. Where indicated cross-linking was performed in the presence of 50 μM U0126, SP600125, or SB202190. White separation lines indicate where lanes from the same blots were joined. (**G**) GPNT ECs were transiently nucleofected with pVCAM-1-luc, reseeded and allowed to grow to confluence (48–72 h posttransfection), and then subjected to ICAM-1 cross-linking (XL) for 6 h or stimulation with IL-1β (IL1β). Where indicated cross-linking was performed in the presence of 50 μM U0126, SP600125, or SB202190. Mean ± SEM luciferase activity (relative to untreated cells) was determined from three independent experiments. *p < 0.05, **p < 0.01, ***p < 0.001.

Next, we investigated changes in mRNA levels of various inflammatory genes in response to ICAM-1 activation. Cross-linking of ICAM-1 for 4 h induced an ∼3-fold increase in VCAM-1, COX-2, and TNF-α transcript levels (Fig. 3A). No changes were detected for CCL2 or ICAM-1. Both in cerebral and dermal MVECs, ICAM-1 activation also led to increases in secreted TNF-α, which were measurable within 4 h and sustained for at least 48 h (Fig. 3B, 3C). Multiplex analysis further revealed significant upregulation of secreted CXCL8, CCL3, and CCL4 but not CXCL10, CCL2, or CCL5 in human cerebral MVECs (Fig. 3D, Supplemental Fig. 2A). Whereas induction of CXCL8 was in part dependent on secreted TNF-α, that of CCL3 and CCL4 was not. ICAM-1 activation did not change production of any of these cytokines in human dermal MVECs (Fig. 3E, Supplemental Fig. 2A). Induction of VCAM-1 expression following ICAM-1 cross-linking in GPNT was also observed at the protein level (Fig. 3F). Pharmacological inhibition of ERK, JNK, or p38 using U0126, SP600125, or SB202190, respectively (see also Supplemental Fig. 2B–D), revealed a strong dependency of VCAM-1 protein induction on ERK and p38 and to a lesser degree JNK activity (Fig. 3F). In agreement, promoter analysis using transiently transfected luciferase reporters showed that ICAM-1 cross-linking enhanced the activity of the human VCAM-1 promoter by 50% and that this response was abolished by inhibition of ERK or p38, but not JNK (Fig. 3G).

**FIGURE 4.**
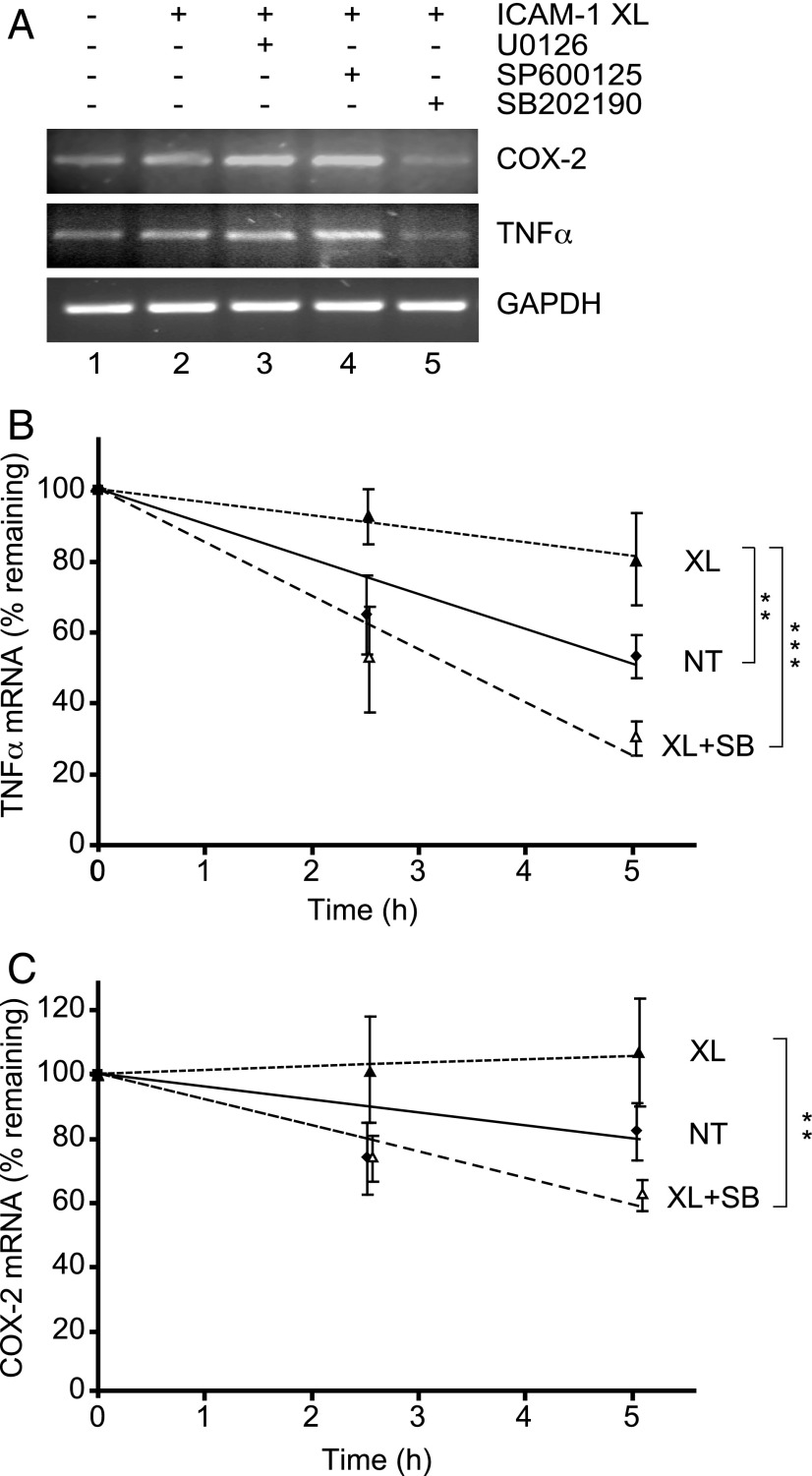
ICAM-1–induced p38-mediated message stabilization. (**A**) Levels of COX-2, TNF-α and GAPDH transcripts were determined by semiquantitative RT-PCR analysis in response to ICAM-1 cross-linking (XL) for 4 h. Where indicated cross-linking was performed in the presence of 50 μM U0126, SP600125, or SB202190. (**B** and **C**) Confluent GPNT cells were serum starved and either left untreated (NT, filled squares) or ICAM-1 cross-linked (XL, filled triangles) in the absence or presence of 50 μM SB202190 (XL + SB, open triangles). Ten micrograms per milliliter actinomycin D was added to block transcriptional activity and total RNA was isolated after 0, 2.5, and 5 h. Subsequently transcript levels of TNF-α and COX-2 were determined by quantitative RT-PCR. The amount of each transcript was quantified by densitometry, normalized, plotted, and analyzed by linear regression and ANOVA. The values are mean ± SEM of seven (TNF-α) and five (COX-2) independent experiments. **p < 0.01, ***p < 0.001.

In contrast to VCAM-1, increases in mRNA levels of TNF-α and COX-2 were only inhibited by the p38 inhibitor (Fig. 4A), suggesting that they could be a result of enhanced message stabilization (20). Message stability in response to ICAM-1 stimulation was measured in transcriptionally blocked GPNT cells. The half-life of TNF-α transcripts, which was 4.9 ± 0.6 h in unstimulated cells, increased to 12.9 ± 3.8 h following ICAM-1 cross-linking (Fig. 4B). This increased stability was sensitive to p38 inhibition. The half-life of COX-2 transcripts, although very stable in the absence of ICAM-1 cross-linking (half-life: 12.2 ± 2.8 h), was also further increased in a p38-sensitive manner following ICAM-1 cross-linking (Fig. 4C). Taken together we concluded that ICAM-1–induced MAPK activation regulated inflammatory gene expression through transcription and posttranscriptional mechanisms.

### Endothelial JNK regulates lymphocyte transmigration

**FIGURE 5.**
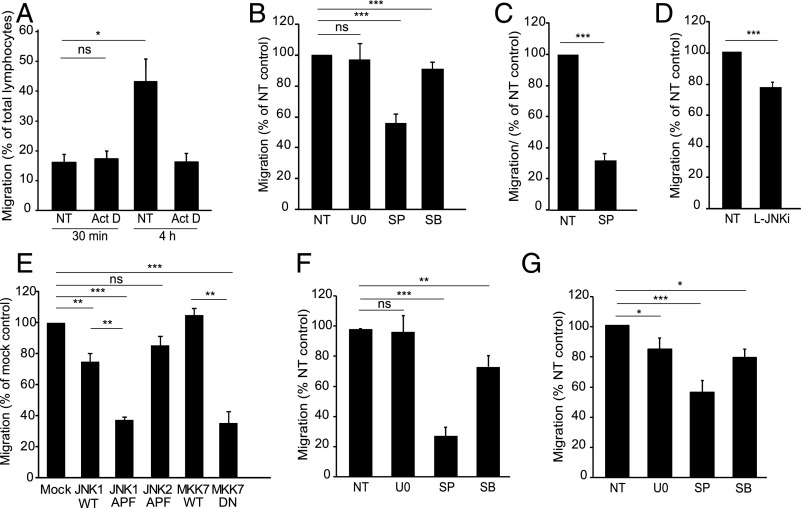
Endothelial JNK regulates lymphocyte TEM. (**A**) GPNT monolayers were pretreated or not with actinomycin D (Act D, 5 μg/ml) and subsequent TEM of Th1 lymphocytes (PAS, see also Supplemental Fig. 3A) measured after 30 min. Whereas Act D did not affect 30 min TEM rates, it inhibited all subsequent TEM events (measured up until 4 h). (**B**) TEM assay as in (A) with the exception that GPNT monolayers were left untreated (NT) or treated with 50 μM U0126 (U0), SP600125 (SP) or SB202190 (SB) for 1 h prior to a 30 min TEM assay. Due to the high washout rate of U0126 from GPNT cells (see Supplemental Fig. 2B), TEM experiments were also conducted with U0126 present throughout. However, even under these conditions TEM was not inhibited (data not shown). (**C**) TEM assay as in (B) except that primary rat brain MVEC were either left untreated (NT) or pretreated with 50 μM SP600125 for 1 h prior to addition of Ag-specific T lymphocytes. (**D**) TEM assay as in (B) with the exception that GPNT were either left untreated (NT) or treated with 1 μM L-JNKi for 1 h prior to the addition of T lymphocytes. (**E**) TEM assay as in (B) except that GPNT cells were transfected with wild-type (WT) or dominant-negative (DN) JNK1, JNK2, or MKK7 48 h before TEM and adhesion were analyzed. (**F** and **G**) TEM assay as in (B) except that TEM of human CD4^+^ cells across hCMEC/D3 (F) or human dermal MVEC (G) was measured following EC pretreatment with 50 μM U0126 (U0), SP600125 (SP), or SB202190 (SB) for 1 h. *p < 0.05, **p < 0.01, ***p < 0.001.

We next investigated whether endothelial gene expression was critical for TEM of CD4^+^ Th1 cells across brain GPNT ECs. Pretreatment of ECs with the transcription inhibitor actinomycin D did not affect lymphocyte TEM, which occurred during the first 30 min of coculture (Fig. 5A). However, all subsequent TEM was inhibited by actinomycin D. We conclude that in this model of lymphocyte TEM initial diapedesis was independent of de novo protein synthesis, whereas at the later time point the majority of TEM required new protein synthesis.

Using U0126 we found that lymphocyte TEM across GPNT did not require endothelial ERK (Fig. 5B). Inhibition of endothelial p38 using SB202190 led to a weak (<10%) inhibition of lymphocyte TEM. In contrast endothelial inhibition of JNK using SP600125 reduced lymphocyte TEM by almost 50%. Significant reduction in lymphocyte TEM with JNK inhibition was also observed in SP600125-treated primary rat brain MVEC (Fig. 5C) or when ECs were pretreated with the peptide inhibitor L-JNKi (Fig. 5D). In all cases the inhibition of endothelial JNK reduced lymphocyte TEM without affecting adhesion (Supplemental Fig. 3B–D). In addition, exogenous expression in GPNTs of dominant-negative but not wild-type JNK1 or MKK7 reduced lymphocyte TEM (but not adhesion) by ∼60% (Fig. 5E, Supplemental Fig. 3E). In contrast exogenous expression of dominant-negative JNK2 did not significantly affect lymphocyte TEM.

To study lymphocyte TEM in human cell models, CD4^+^ cells isolated from human blood were allowed to transmigrate across either cerebral hCMEC/D3 cells or primary human dermal MVECs. MAPK dependency of TEM across human cerebral MVECs mirrored that observed for rat Th1/GPNT cocultures: TEM was very sensitive to inhibition of endothelial JNK, to a much lesser degree to inhibition of p38, and not at all to ERK inhibition (Fig. 5F). In contrast, TEM of lymphocytes across human dermal MVECs was sensitive (albeit to a lesser overall degree) to inhibition of JNK and p38 as well as ERK (Fig. 5G), illustrating clear mechanistic differences of TEM in cerebral and peripheral MVECs.

### Src, Rho, and PKC are involved in ICAM-1–mediated MAPK activation

**FIGURE 6.**
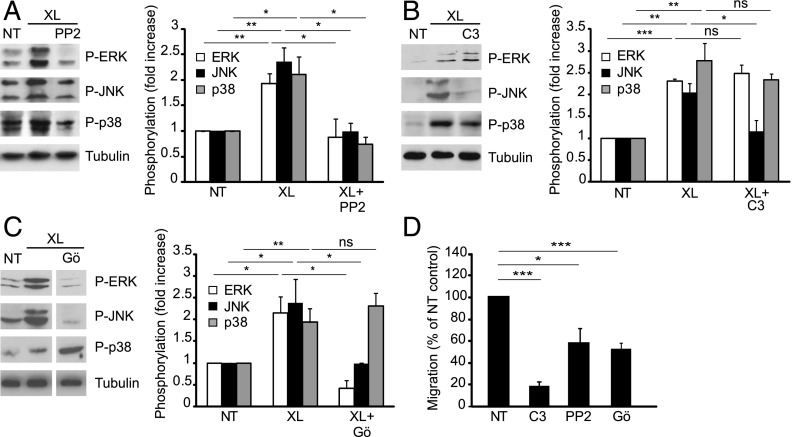
Role of Src, Rho GTPase, and PKC in ICAM-1–mediated MAPK activation and lymphocyte TEM. Postconfluent, serum-starved GPNT cells were either left untreated (NT) or pretreated with 10 μM PP2 for 30 min (**A**), 10 μg/ml C3 transferase for 12 h (**B**), or 20 μM Gö6983 (Gö) for 30 min (**C**). In (C) white separation lines indicate where lanes from the same blots were joined. Where indicated EC monolayers were subjected to ICAM-1 cross-linking (XL) for 10 min. MAPK phosphorylation was then analyzed and quantified as described for Fig. 1. Results similar to those shown with PP2 were also found with 10 μM SU6656 (data not shown). MAPK levels were not significantly affected by any of the pretreatments (Supplemental Fig. 1C). (**D**) GPNT EC monolayers were pretreated with 10 μM PP2 for 1 h, 10 μg/ml C3 transferase for 16 h, or 20 μM Gö6983 (Gö) for 1 h prior to analysis of lymphocyte TEM. *p < 0.05, **p < 0.01, ***p < 0.001.

Established endothelial ICAM-1 signaling effectors include Src, Rho GTPase, and PKC (2). To examine how MAPK activation was integrated into these signaling pathways, we used pharmacological inhibitors in GPNT ECs. Inhibition of Src using PP2 abolished ICAM-1–induced phosphorylation of all three endothelial MAPKs (Fig. 6A). Inhibiting Rho GTPase using C3 transferase only affected the phosphorylation of JNK, having no effect on the ICAM-1–mediated increase in ERK or p38 phosphorylation (Fig. 6B). A different pattern of inhibition was elicited by blocking PKC with Gö6983, whereby it prevented the activation of ERK and JNK, but had no effect on p38 (Fig. 6C). None of the treatments affected overall levels of MAPKs (Supplemental Fig. 1C). Inhibition of either Src, Rho GTPase, or PKC in GPNT ECs also resulted in significant inhibition of lymphocyte TEM without affecting adhesion to EC monolayers (Fig. 6D, Supplemental Fig. 3F), in agreement with published data (8, 9, 12, 31) and indicating that they acted in concert with JNK in regulating TEM.

### Phosphorylation of paxillin via a JNK-dependent pathway regulates lymphocyte TEM and VE-cad internalization in cerebral MVECs

**FIGURE 7.**
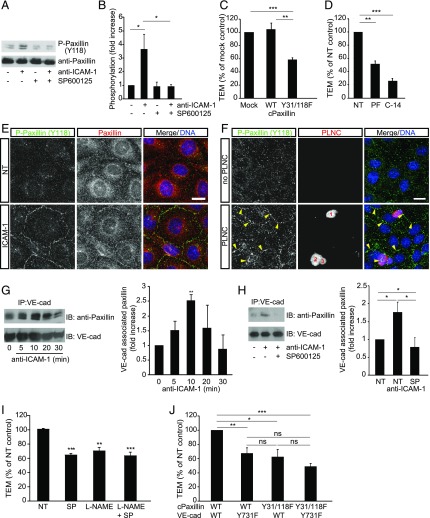
Endothelial JNK mediates F-actin rearrangements and paxillin phosphorylation in the regulation of lymphocyte TEM. (**A**) GPNT cells were pretreated without or with 50 μM SP600125 for 1 h before 30 min ICAM-1 ligation. Cell lysates were analyzed by immunoblots using anti–phospho-Y118 paxillin and anti-paxillin Abs. (**B**) Densitometric quantification of paxillin phosphorylation from three independent experiments as shown in (A). (**C**) Expression of phosphorylation-deficient Y31F/Y118F (Y31/118F) significantly inhibited lymphocyte TEM (measured as described in Fig. 5E). (**D**) Pretreatment of GPNT with the FAK inhibitors PF573228 (PF, 10 μM) or FAK inhibitor 14 (C-14, 50 μM) for 1 h led to inhibition of TEM (measured as described in Fig. 5B). (**E**) Postconfluent, serum-starved GPNT ECs were subjected to ICAM-1 ligation for 30 min, and then fixed and stained for phospho-Y118 paxillin (green) and total paxillin (red). Shown are representative confocal micrographs. (**F**) Postconfluent GPNT monolayers were left untreated (upper panels), or PLNCs (∼5 PLNCs per EC) were added (lower panels) and allowed to adhere for 30 min. Cultures were then vigorously washed to remove all loosely attached PLNCs, fixed and stained for phospho-paxillin (red) and DNA (blue), and analyzed by confocal microscopy. Numbers indicate three individual adherent T cells. Arrowheads indicate phospho-paxillin in cell-cell contact strands. Scale bars, 10 μm. (**G**) ICAM-1 was ligated in postconfluent, serum-starved GPNT cells for the indicated times before the cells were lysed and VE-cad immunoprecipitated. Representative immunoblots show the level of paxillin and VE-cad found in immunoprecipitates. Densitometric analysis (mean ± SEM) from five such experiments is shown on the right. (**H**) As in (G) with the exception that postconfluent GPNT were pretreated with 50 μM SP600125 for 1 h and subjected to ICAM-1 ligation for 15 min before analysis; quantification was from four independent experiments. (**I**) TEM assay as described in Fig. 5B except that GPNTs were pretreated with 50 μM SP600125 (SP), 1 mM L-NAME, or both as indicated. (**J**) TEM assay as described in Fig. 5E except that GPNTs were cotransfected with combinations of plasmids encoding wild-type or phosphorylation-deficient Y31F/Y118F (Y31/118F) paxillin and wild-type or phosphorylation-deficient Y731F mouse VE-cad as indicated. *p < 0.05, **p < 0.01, ***p < 0.001.

We next focused on the key role of JNK in lymphocyte TEM across cerebral MVECs. Specifically, we investigated the relationship between ICAM-1–mediated JNK activation and events critical to TEM, namely endothelial actin reorganization as well as paxillin and VE-cad phosphorylation (3, 9, 12, 13). Ligation of ICAM-1 in GPNTs induced F-actin changes, with cortical bundles dominating the cellular architecture after ∼20 min (Supplemental Fig. 3K). Inhibition of JNK, but not ERK or p38, prevented this cortical actin induction. Ab-mediated ligation of ICAM-1 also induced robust phosphorylation of paxillin on Y118 (Fig. 7A, 7B), which was abolished by inhibition of JNK. Exogenous expression of Y31F/Y118F phosphorylation-deficient paxillin in ECs inhibited lymphocyte TEM significantly by 42% (Fig. 7C), but had no effect on adhesion (Supplemental Fig. 3G). In agreement, we found that inhibition of endothelial FAK, which phosphorylates paxillin on Y118 (14), also significantly reduced lymphocyte TEM (Fig. 7D, Supplemental Fig. 3H). ICAM-1–induced phosphorylation of paxillin occurred in the cell-cell contact areas of GPNT (Fig. 7E), suggesting that ICAM-1–induced JNK-paxillin signaling was involved in regulating endothelial junctions. Continuous strands of phospho-paxillin staining could also be detected in the vicinity of adherent PLNCs (Fig. 7F). These data prompted an analysis of the association of paxillin with VE-cad during ICAM-1 activation and TEM. As shown in Fig. 7G, ICAM-1 activation led to enhanced association of VE-cad with paxillin, reaching peak levels after around 10 min. This ICAM-1–induced association of VE-cad and paxillin could be completely blocked by inhibition of JNK (Fig. 7H). In turn, MAPK inhibition did not affect ICAM-1–induced VE-cad phosphorylation (Supplemental Fig. 3L).

The cooperation of paxillin and VE-cad was further confirmed in TEM experiments of CD4^+^ Th1 lymphocytes across GPNT cell monolayers. Inhibition of the paxillin activation pathway through either SP600125 pretreatment or expression of Y31F/Y118F paxillin led to TEM inhibition that was similar to that when the VE-cad pathway was inhibited using either L-NAME pretreatment (10) or expression of Y731F VE-cad (Fig. 7I, 7J). Combination of treatments to inhibit both pathways did not result in additive effects, indicating that paxillin and VE-cad phosphorylation functionally converged during TEM. Taken together these data suggested that ICAM-1/JNK activation mediated phosphorylation of paxillin and its association with VE-cad to regulate lymphocyte TEM.

**FIGURE 8.**
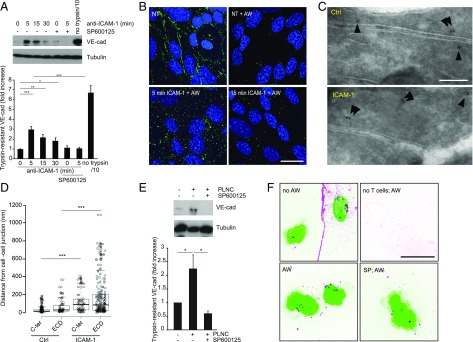
ICAM-1 mediates VE-cad internalization in a JNK-dependent manner. (**A**) ICAM-1 was ligated in postconfluent, serum-starved GPNT. At indicated times, cells were transferred to ice to stop endocytosis, treated with trypsin, and lysed, and the level of trypsin-resistant VE-cad determined by immunoblot analysis. Shown are representative immunoblots and densitometric quantification of trypsin-resistant (i.e., internalized) VE-cad in comparison with cellular tubulin content. A 10th of nontreated control cell extract was loaded to reveal total VE-cad content (no trypsin/10). Where indicated, cells were pretreated with 50 μM SP600125. (**B**) VE-cad endocytosis was visualized by internalization of a FITC-labeled anti–VE-cad Ab (green) in postconfluent, serum-starved hCMEC/D3 cells. Cells were left untreated or treated with anti–ICAM-1 (5 μg/ml) for the indicated times. Where indicated extracellular Ab was removed by acid wash (AW). Subsequently cells were fixed, their nuclei counterstained (blue), and analyzed by confocal microscopy. Scale bar, 10 μm. (**C**) Cryo-immuno–EM of VE-cad distribution in control (Ctrl) and anti–ICAM-1 stimulated (5 min) hCMEC/D3 cultures. Shown are interendothelial junction areas with the two abutting plasma membranes. Arrowheads point out gold labeled VE-cad, which in control cells was found predominantly associated with the plasmalemmal membrane (within 20 nm, i.e., the distance expected by the primary and the secondary bridging Ab) (23). (**D**) Distances measured from cell-cell junction for VE-cad gold particles as determined from three independent preparations as shown in (C). (**E**) As in (A) with the exception that postconfluent, serum-starved GPNT were cocultured with PLNCs (∼5 PLNCs per EC) for 15 min. (**F**) hCMEC/D3 were cocultured with human CD4^+^ lymphocytes (green) for 30 min. VE-cad (magenta) distribution and endocytosis was visualized as described in (B) by Ab labeling and acid wash before fixation and confocal microscopy. Scale bar, 10 μm. *p < 0.05, **p < 0.01, ***p < 0.001.

**FIGURE 9.**
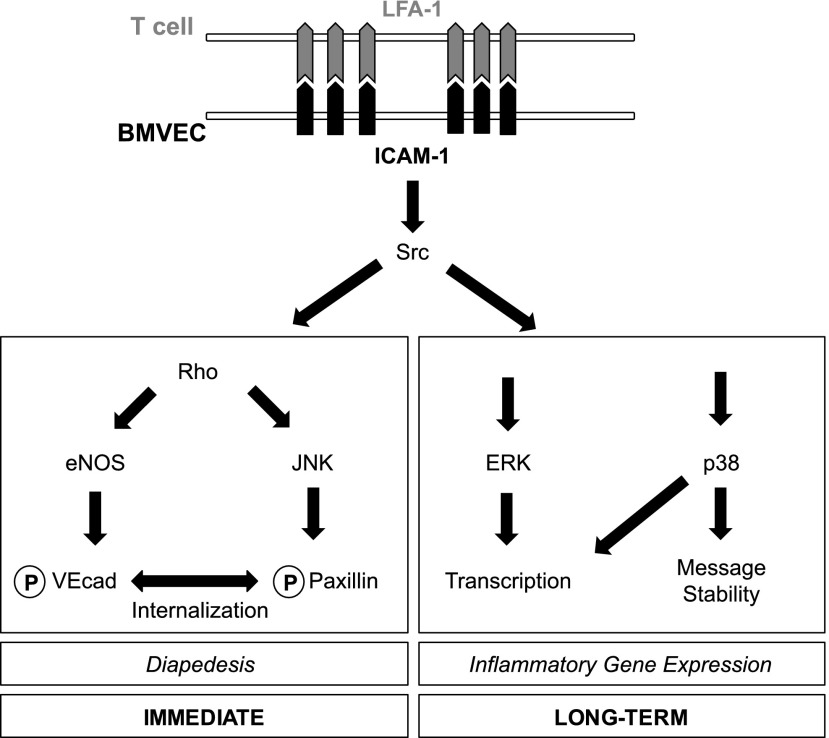
Proposed signaling networks in brain MVEC downstream of ICAM-1. Circled P indicates protein phosphorylation.

VE-cad has been shown to internalize during TEM (32). Here, we show that ICAM-1 stimulation was sufficient to induce VE-cad internalization. ICAM-1 stimulation increased the proportion of VE-cad in GPNT cells that was resistant to trypsin treatment, indicating it had been removed from the plasmalemmal surface and internalized (Fig. 8A). Trypsin-resistant VE-cad increased ∼3-fold within 5 min of anti–ICAM-1 Ab addition (representing ∼4% of all cellular VE-cad). ICAM-1 stimulation also enhanced endocytosis of a VE-cad–specific Ab in hCMEC/D3 cells, with peak activity occurring at around 5–10 min (Fig. 8B). Furthermore, ultrastructural inspection by cryo-immuno–electron microscopy (EM) revealed a significant relocalization of VE-cad from the junction to the cell interior by a distance of at least 50 nm (Fig. 8C, 8D). Importantly within the context of our work, ICAM-1–mediated VE-cad internalization was SP600125-sensitive, indicating that it was under the control of JNK signaling (Fig. 8A). VE-cad internalization also occurred in response to lymphocyte adhering to brain ECs. Coculturing GPNT with nonmigratory PLNC for 15 min significantly increased trypsin-resistant VE-cad in SP600125-sensitive manner (Fig. 8E). Microscopic analysis of cocultures of hCMEC/D3 with human CD4^+^ cells revealed SP600125-sensitive VE-cad endocytosis which occurred most strongly within the area of the adhesion footprint of the lymphocyte (Fig. 8F). Taken together, these results indicate that ICAM-1 via JNK and paxillin regulated VE-cad internalization during TEM (Fig. 9).

## Discussion

Endothelial ICAM-1 stimulation induces or enhances a variety of inflammatory responses including vascular permeability, inflammatory gene expression, and the licensing of leukocyte TEM (2, 5, 6, 15, 16). However, the underlying signal transduction networks responsible for these diverse effects are incompletely understood. Here, we show interconnectivity of many previously recognized components of ICAM-1 signaling through MAPKs, and attribute diapedesis-related and diapedesis-unrelated functions (Fig. 9). The MAPKs ERK, JNK, and p38 were activated in response to lymphocyte adhesion, with signaling via ICAM-1 contributing almost 50%. This is in line with previous reports of MAPKs as important effector molecules of endothelial ICAM-1 signaling (7, 17, 21) and of other EC surface receptors (5). As in previous reports MAPK activation was observed by receptor cross-linking (consisting of a 30 min incubation with primary Ab followed by clustering with a secondary Ab). Additionally, simple ICAM-1 ligation (10) (where only the primary Ab is used) induced MAPKs with the only notable difference being the length of their activation.

The balance of MAPK activation determines the inflammatory state of the endothelium (19). In brain MVECs, MAPKs regulated inflammatory gene expression in two ways. On the one hand, they induced transcription of genes such as VCAM-1 consistent with their ability to control the activity of transcription factors such as AP-1 and NF-κB, binding sites for which are found in the VCAM-1 promoter (33, 34). Thus ICAM-1 signaling regulated microvascular endothelial gene transcription in a way similar to that reported for macrovascular HUVECs (17, 35). On the other hand, ICAM-1 stimulation regulated gene expression in previously unrecognized manner posttranscriptionally by significantly lengthening the half-life of mRNAs encoding TNF-α and COX-2. This is likely to occur via binding of proteins such as tristetraprolin, human Ag R, and KH-type splicing regulatory protein to AU-rich motifs in the 3′ region of mRNAs encoding cytokines such as COX-2 and TNF-α (20, 36). Importantly, mRNA stabilization involving AU-rich motifs is heavily dependent on p38 activity and this is exactly what we found for ICAM-1–induced stabilization of messages. ICAM-1 stimulation induced inflammatory cytokines relevant to neuroinflammation (28), including TNF-α, CXCL8, CCL3, CCL4, and VCAM-1 but not CXCL10, CCL2, or CCL5. TNF-α induction and secretion contributed in part to ICAM-1–mediated CXCL8 upregulation in cerebral ECs. In dermal ECs ICAM-1 also enhanced TNF-α secretion, albeit with a slower time course, and there were no changes of the other cytokines tested, indicating that ICAM-1 signaling induces fundamentally different responses in different vascular beds. In prototypical neuroinflammatory processes, such as multiple sclerosis, a wide range of chemokines is found expressed by CNS parenchymal cells or infiltrated leukocytes but importantly also ECs in the vicinity of lesions (28). Chemokine expression such as CXCL8 by cerebral ECs themselves appears to be important to bypass restrictions imposed by the blood-brain barrier to take up, transport, and present parenchymally produced factors. Although chemokine production of cerebral MVECs in response to cytokine stimulation has been recognized for some time (28), our data indicates that TEM alone also contributes to shaping the inflammatory vascular fingerprint in the immediate vicinity of a TEM event in the brain. Multiple types of leukocyte are capable of crossing the same region of the vascular endothelium (37). For multiple sclerosis it is generally accepted that infiltration of autoantigen-specific T lymphocytes [such as our PAS cells, which upon adoptive transfer induce the model disease experimental autoimmune encephalomyelitis (26)] precedes that of the main effector cells (mainly monocytes but also B lymphocytes and neutrophils), which then mediate devastating demyelination (38). Thus altered EC gene expression in response to ICAM-1 ligation may contribute to subsequent attraction of other leukocytes to sites of inflammation. These may include neutrophils and monocytes given the strong induction of CXCL8 and CCL3 and 4 (39, 40). Alternatively, because lymphocytes have also been reported to express receptors for these chemokines (40–43) initial T cell TEM may attract more T cells to the permissive site identified by pioneering cells. Indeed, lymphocyte TEM within a physiologically relevant timeframe of 30 min (44) did not require gene transcription in cerebral ECs. However, subsequent TEM events clearly needed endothelial de novo RNA synthesis, suggesting that either turnover of the TEM machinery occurred at later time points or that a secondary wave of lymphocytes required the expression of certain EC molecules (such as chemokines or VCAM-1). To our knowledge this is a novel paradigm likely to have been overlooked in the majority of previous studies, which measured rates after at least 4 h of coculture (e.g., 9, 45, 46).

Among the three MAPKs studied, endothelial JNK was most important for facilitating lymphocyte TEM across both cerebral and noncerebral ECs. The contribution of p38 was much weaker. Significantly, in dermal MVECs where ERK was also much more strongly activated by ICAM-1, ERK was also required for TEM indicating that TEM differed at least in part across different vascular beds. Given that both ERK and p38 have been reported to regulate cytokine-stimulated TEM of neutrophils across noncerebral ECs (21, 47), MAPK requirements may depend on the tissue context of the endothelium, its activation state, and/or the leukocyte subtype undergoing TEM. Focusing further on the novel role of JNK during TEM, we corroborated its role in cerebral MVECs by expressing dominant-negatives of JNK1, JNK2, and MKK7. These experiments also indicated that JNK1 rather than JNK2 regulated lymphocyte TEM. JNK activation occurred downstream of Src, Rho GTPase, and PKC, which are all involved in regulating the TEM of various leukocyte subtypes across CNS and non-CNS endothelium (2, 7–9, 31). Crucially, the key TEM regulator Rho GTPase was not required for the activation of ERK or p38, further underlining that these two MAPKs played a minor or no role for TEM across cerebral ECs. Taken together, these data firmly place JNK but not ERK or p38 at the center of putative TEM licensing pathway(s) in the neural vasculature (Fig. 9).

Paxillin phosphorylation downstream of ICAM-1 stimulation has long been known (7). Recently a critical role for this cortical scaffold protein and its upstream regulator FAK has also been demonstrated in TEM of neutrophils (13). Our study validated the involvement of paxillin/FAK for TEM across cerebral MVECs and provided additional mechanistical details. ICAM-1–induced phosphorylation of paxillin on Y118 was critical for TEM and this was dependent on JNK activity. Notably, TEM was dependent to the same extent on endothelial JNK activation as it was on Y31/Y118 paxillin phosphorylation, suggesting that the latter was a key effector process of endothelial JNK activation. Tyrosine phosphorylation of paxillin is often dependent on prior JNK-mediated serine phosphorylation (48). In agreement, we found that expression of paxillin, phosphorylation-deficient on the JNK-specific site S178, was as effective in inhibiting TEM as was the tyrosine phosphorylation mutant (Supplemental Fig. 3I). A similar JNK-dependent pathway has been shown to regulate paxillin in normal rat kidney epithelial cells, where it is involved in wound closure (49). Paxillin is mainly involved in the dynamic regulation of focal adhesions and integrin-mediated cell motility (14, 50). In cerebral MVECs, ICAM-1 stimulation or lymphocyte adhesion resulted in the accumulation of tyrosine phosphorylated paxillin at cell-cell junctions and association with VE-cad complexes. Thus the current study adds to an increasing number of reports of interactions of classical focal adhesion proteins with endothelial AJs in the regulation of endothelial barrier function (51–54).

TEM licensing and the regulation of endothelial barrier function are mechanistically related. Disruption or loosening of the AJs has been observed during TEM in vitro (55). Likewise, vascular permeability is also accompanied by AJ disassembly (56) and importantly can be induced by ICAM-1 stimulation and adherent leukocytes (3, 6). In agreement, phosphorylation of VE-cad and its subsequent internalization are hallmarks of both processes (3, 12, 30, 32, 56). Here, we show that ICAM-1-JNK-paxillin signaling induced VE-cad internalization. During TEM, phosphorylation and internalization may target VE-cad for degradation via an endosome-lysosomal pathway and AJs may disassemble as a consequence. Alternatively, VE-cad internalization may be part of dynamic rearrangements during the loosening of paracellular junctions. Thus paracellular junctions are regulated by ICAM-1 during TEM through at least two converging signaling pathways. First, VE-cad phosphorylation is regulated through AMP–endothelial NO synthase (10). Second, we show here that JNK-mediated association with paxillin led to VE-cad internalization. Overall, our work lends further evidence to a central role of VE-cad as a molecular and cellular barrier function of ECs but also assigns a completely novel role to paxillin in this process.

When examined by cryo-immuno–EM, much of the internalized VE-cad was found in close proximity to the junction, suggesting that it originated from the junction area itself. However, ICAM-1 stimulation clearly induced internalization throughout the EC. Furthermore, in agreement with published data (32), internalized VE-cad was detectable within the broader adhesion footprint of the lymphocyte often being a considerable distance from the paracellular junction area, raising the possibility that endosomal VE-cad traveled much further or originated from nonjunctional plasmalemmal membrane areas. Our studies focused on events during lymphocyte adhesion and did not allow correlations to be made between VE-cad internalization and the subsequent TEM route. However, because all adherent lymphocytes appeared to be associated with some internalized VE-cad and because TEM across brain ECs is both paracellular and transcellular (57, 58), VE-cad internalization is likely to be relevant to both TEM routes.

In conclusion, the concomitant activation of MAPKs is central to ICAM-1–mediated signaling in cerebral (Fig. 9) but also in other vascular MVECs. Unsurprisingly, divergence was observed in signaling and in downstream effector functions, with all three MAPKs regulating transcription, solely p38 regulating posttranscriptional mRNA stabilization, and mainly JNK regulating lymphocyte TEM. Future work should determine 1) if a TEM event can prime subsequent TEM events through altered endothelial gene expression; and 2) the role and endosomal trafficking of vesicular VE-cad in the regulation of paracellular and transcellular TEM (59).

## Supplementary Material

Data Supplement
