# Supplementary material for: Endothelial MAPKs Direct ICAM-1 Signaling to Divergent Inflammatory Functions
Source: J Immunol. 2017 Apr 3;198(10):4074–85. doi: 10.4049/jimmunol.1600823 (PMC5421301; doi:10.4049/jimmunol.1600823)
Supplement: Data Supplement [file JI_1600823.zip › JI_1600823_Supplemental_Material_1.pdf]

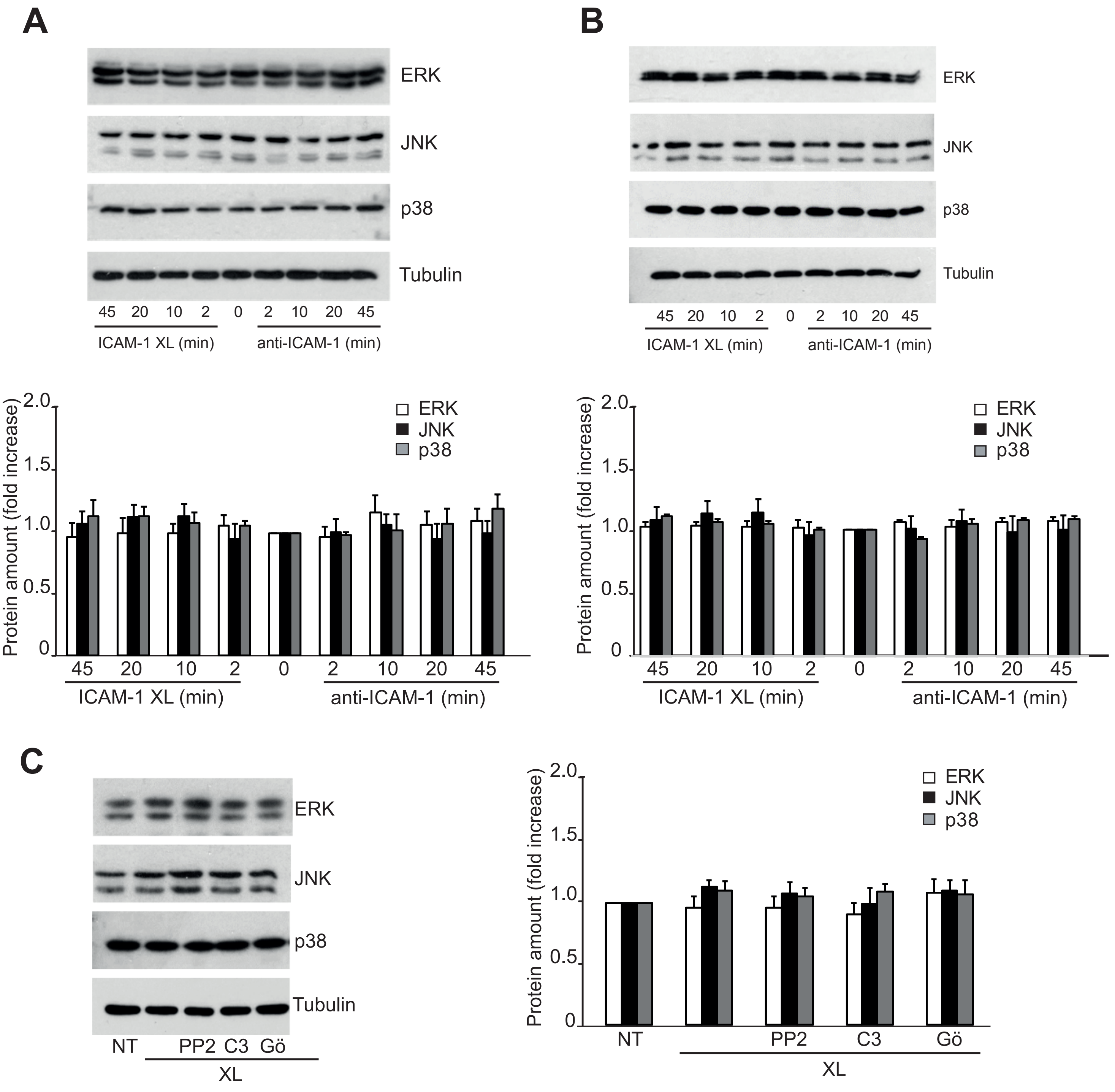

Figure S1. (**A**) GPNT BMVEC were subjected to ICAM-1 cross-linking (XL) for the indicated length of time and total MAPK analyzed. Representative results and quantification of total MAPK (normalized means ± SEM) from three independent experiments are shown. (**B**) As in (A) except that the XL was performed on hDMEC. (**C**) Post-confluent, serum starved GPNT cells were either left untreated (NT) or pretreated with 10 μM PP2 for 30 min (A), 10 μg/ml C3 transferase for 12 h (B) or 20 μM Gö6983 (Gö) for 30 min. Where indicated EC monolayers were subjected to ICAM-1 cross-linking (XL) for 10 min. Basal MAPK activity was then analyzed and quantified as in (A).

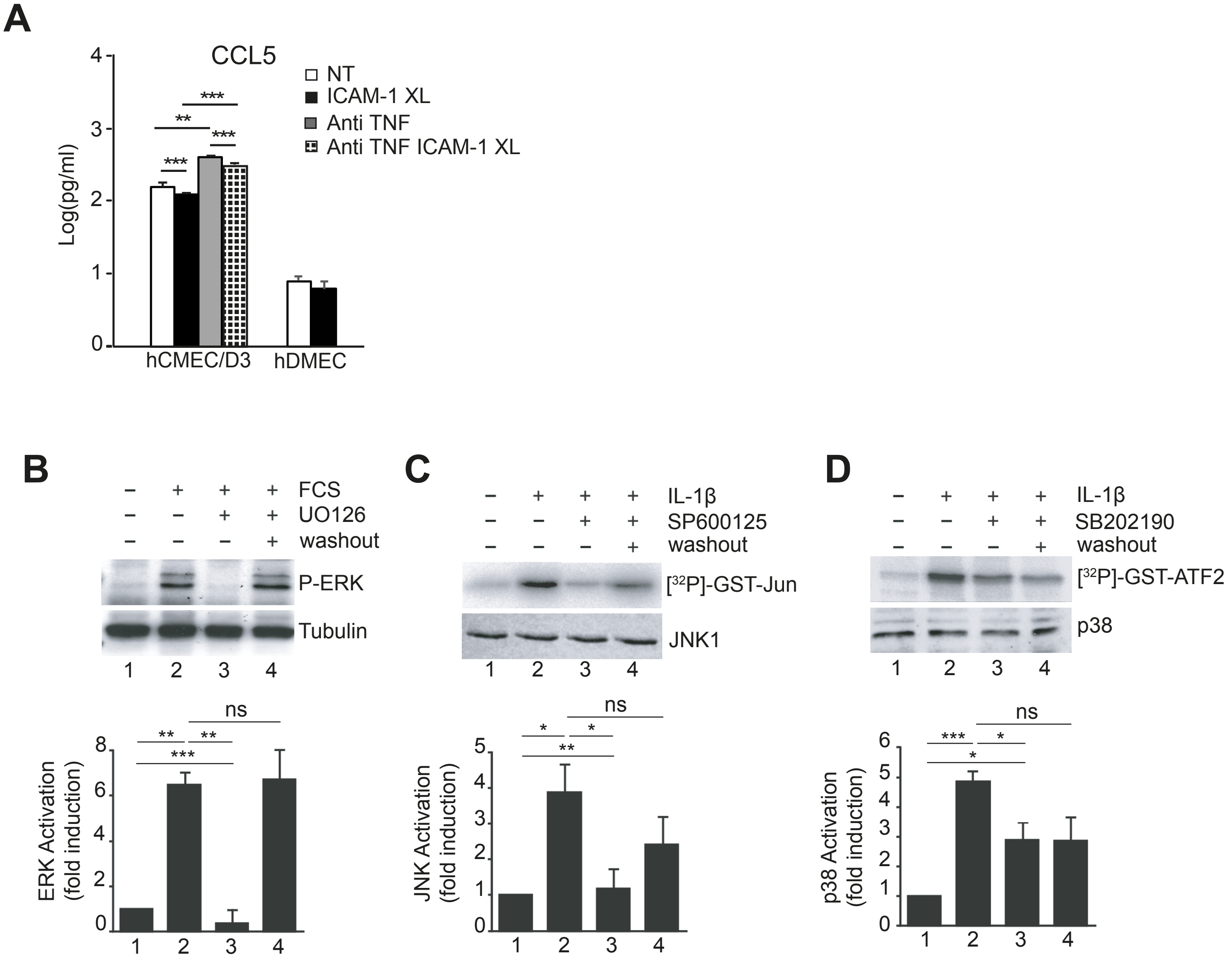

Figure S2. (**A**) hCMEC/D3 or hDMEC were left untreated (NT) or subjected to ICAM-1 cross-linking for 24 h (XL). The concentration of CCL5 in the supernatant was measured by Multi-Analyte Flow Assay. Where indicated anti-TNFα (1µg/ml) was included during the stimulation period to determine if altered chemokine secretion was a consequence of TNFα induction. Shown are mean concentrations (± SEM) of chemokines in the culture supernatant as determined from 3 independent experiments.
(**B**) Sub-confluent, serum starved GPNT cells were either left untreated or treated with 50 µM U0126 for 1 h. Cells were then stimulated using 10% FCS for 10 min as indicated. Alternatively, U0126 was washed off and the cells incubated for further 30 min in the absence of the drug before serum stimulation (washout). Cells were lysed in boiling SDS-sample buffer and analyzed by immunoblotting with anti-phospho-ERK antibodies and anti-tubulin as loading control. Similar results were achieved using U0126 at concentration up to 100 µM or the alternative inhibitor PD184352 at 50 µM (data not shown). (**C, D**) GPNT were either left untreated or subjected to 50 µM SP600125 (C) or SB2021290 (D) for 1 h as indicated. Cells were then stimulated with 100 U/ml IL-1β for 10 min. Alternatively, the drugs were washed out as described in (B) before cytokine stimulation (washout). Subsequently, JNK (C) and p38 (D) were immunoprecipitated and subjected to *in vitro* kinase assays using GST-Jun and GST-ATF2 as substrate, respectively, or immunoblotted for JNK1 or p38. Relative kinase activities were determined by densitometric analysis of autoradiographs normalized to the total kinase present. All values are means ± SEM from three independent experiments.

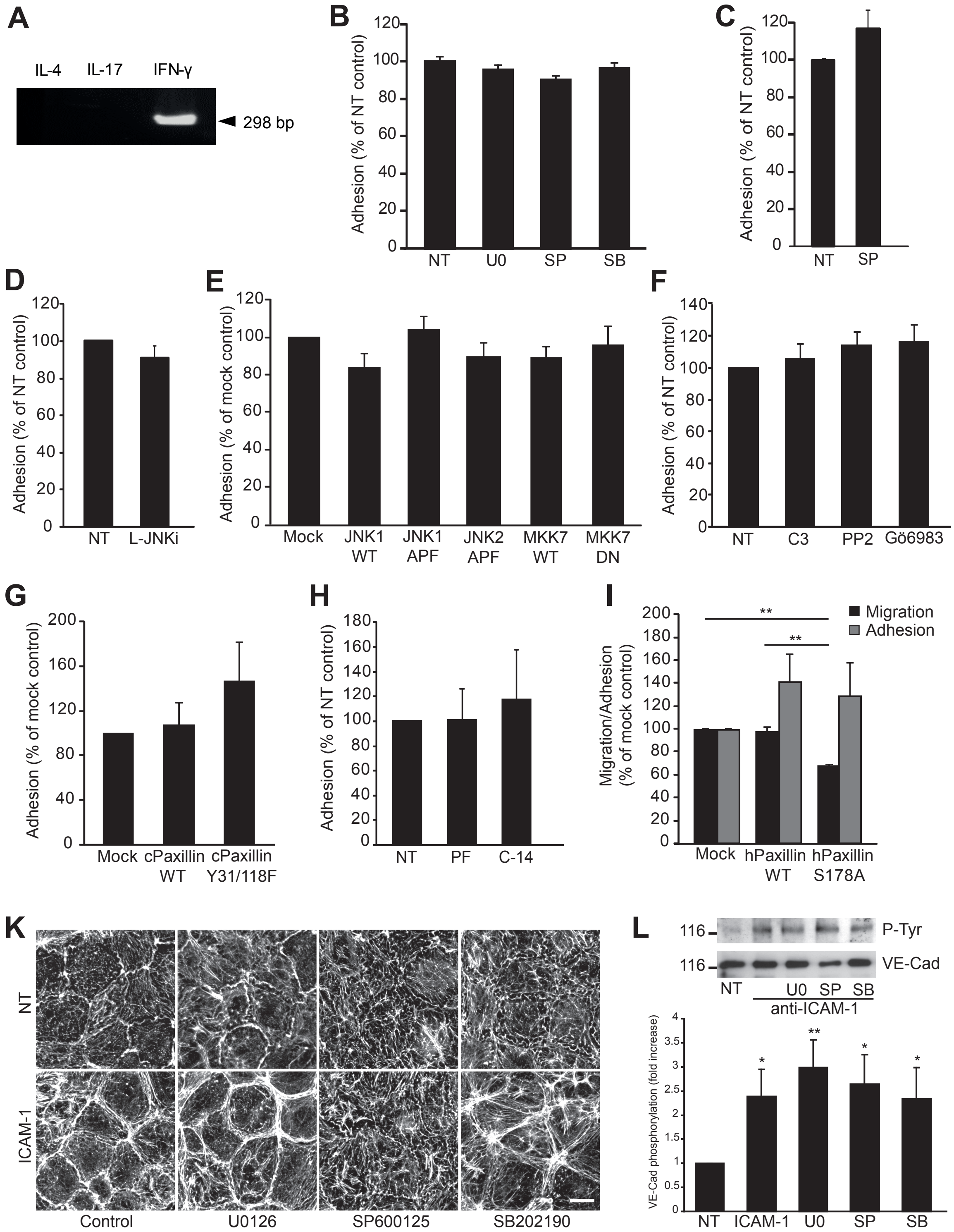

Figure S3. (**A**) Total RNA was isolated from PAS and analyzed by semi-quantitative RT-PCR using specific primers for IL-4, IL-17 and IFN-γ. (**B-H**) Lymphocyte adhesion to GPNT, See Figures 5 and 7 for details of treatments. (**I**) 10 µg of each CMV expression plasmid encoding human paxillin, either wild-type or phosphorylation-deficient in S178 (S178A), were transfected into GPNT BMVECs and lymphocyte adhesion and TEM assessed. (**K**) ICAM-1 ligation (20 min) induced cortical actin bundle formation, which was sensitive to inhibition of JNK but not ERK or p38. (**L**) Post-confluent GPNT were either left untreated or pre-treated with 50 µM U0126 (U0), SP600125 (SP) or SB202190 (SB) for 1 h prior to ICAM-1 ligation (for 20 min). Subsequently, VE-cad was immunoprecipitated and -blotted using anti-phospho-tyrosine antibodies. MAPK inhibition did not affect tyrosine phosphorylation of VE-cad.

Table S1 Oligonucleotide pairs and annealing temperatures used for PCR amplification of the indicated cDNA.

Gene	Primers	Annealing Temperature
VCAM-1	5'-ATGCCTGGGAAGATGGTCGC-3'<br>5'-CAGGAGCCAAACACTTGACC-3'	60ºC
TNFα	5'-TGCCTCAGCCTCTTCTCATT-3'<br>5'-TGTGGGTGAGGAGCACATAG-3'	57ºC
COX-2	5'-ATCCTGAGTGGGATGACGAG-3'<br>5'-CTGCTTGTACAGCGATTGGA-3'	55ºC
RANTES	5'-GTGCCCACGTGAAGGAGTAT-3'<br>5'-AGCCTGTGAAGAGCACACCT-3'	60ºC
ICAM-1	5'-CCTGTTTCCTGCCTCTGAAG-3'<br>5'-CCTGGGGGAAGTACTGTTCA-3'	60ºC
MCP-1	5'-ATGCAGTTAATGCCCCACTC-3'<br>5'-TTCCTTATTGGGGTCAGCAC-3'	57ºC
c-Fos	5'-CCGACTCCTTCTCCAGCAT-3'<br>5'-CGCAGACTTCTCGTCTTCAA-3'	50ºC
c-jun	5'-ACGACCTTCTACGACGATGC -3'<br>5'-GACACTGGGCAGCGTATTCT -3'	50ºC
GAPDH	5'-ACCACAGTCCATGCCATCAC-3'<br>5'-TCCACCACCCTGTTGCTGTA-3'	55ºC

IL-4	5’-TGCACCGAGATGTTTGTACC-3’<br><br>5’-AGGACATGGAAGTGCAGGAC-3’	60ºC
IL-17	5’-CTTCACCCTGGACTCTGAGC-3’<br><br>5’-AAACGCAGGGGTTTCTTAGG-3'	60ºC
IFN-γ	5’-AGGAAAGAGCCTCCTCTTGG-3’<br><br>5’-TTCTGGTGACAGCTGGTGAA-3’	60ºC
